# Importance of resection margin after resection of colorectal liver metastases in the era of modern chemotherapy: population-based cohort study

**DOI:** 10.1093/bjsopen/zrae035

**Published:** 2024-05-08

**Authors:** Emil Östrand, Jenny Rystedt, Jennie Engstrand, Petter Frühling, Oskar Hemmingsson, Per Sandström, Malin Sternby Eilard, Bobby Tingstedt, Pamela Buchwald

**Affiliations:** Department of Surgery, Skåne University Hospital, Lund, Sweden; Department of Clinical Sciences Lund, Lund University, Lund, Sweden; Department of Surgery, Skåne University Hospital, Lund, Sweden; Department of Clinical Sciences Lund, Lund University, Lund, Sweden; Division of Surgery, Karolinska University Hospital, Stockholm, Sweden; Department of Clinical Science, Intervention and Technology (CLINTEC), Karolinska Institute, Stockholm, Sweden; Department of Surgery, Akademiska University Hospital, Uppsala, Sweden; Department of Surgical Sciences, Uppsala University, Uppsala, Sweden; Department of Surgical and Perioperative Sciences, Umeå University, Umeå, Sweden; Department of Surgery, Linköping University Hospital, Linköping, Sweden; Department of Clinical and Experimental Medicine Sciences, Linköping University, Linköping, Sweden; Department of Transplantation and Liver Surgery, Sahlgrenska University Hospital, Gothenburg, Sweden; Department of Clinical Sciences, Sahlgrenska Academy, University of Gothenburg, Gothenburg, Sweden; Department of Surgery, Skåne University Hospital, Lund, Sweden; Department of Clinical Sciences Lund, Lund University, Lund, Sweden; Department of Surgery, Skåne University Hospital, Malmö, Sweden; Department of Clinical Sciences Malmö, Lund University, Lund, Sweden

## Abstract

**Background:**

Resection margin has been associated with overall survival following liver resection for colorectal liver metastasis. The aim of this study was to examine how resection margins of 0.0 mm, 0.1–0.9 mm and ≥1 mm influence overall survival in patients resected for colorectal liver metastasis in a time of modern perioperative chemotherapy and surgery.

**Methods:**

Using data from the national registries Swedish Colorectal Cancer Registry and Swedish National Quality Registry for Liver, Bile Duct and Gallbladder Cancer, patients that had liver resections for colorectal liver metastasis between 2009 and 2013 were included. In patients with a narrow or unknown surgical margin the original pathological reports were re-reviewed. Factors influencing overall survival were analysed using a Cox proportional hazard model.

**Results:**

A total of 754 patients had a known margin status, of which 133 (17.6%) patients had a resection margin <1 mm. The overall survival in patients with a margin of 0 mm or 0.1–0.9 mm was 42 (95% c.i. 31 to 53) and 48 (95% c.i. 35 to 62) months respectively, compared with 75 (95% c.i. 65 to 85) for patients with ≥1 mm margin, *P* < 0.001. Margins of 0 mm or 0.1–0.9 mm were associated with poor overall survival in the multivariable analysis, HR 1.413 (95% c.i. 1.030 to 1.939), *P* = 0.032, and 1.399 (95% c.i. 1.025 to 1.910), *P* = 0.034, respectively.

**Conclusions:**

Despite modern chemotherapy the resection margin is still an important factor for the survival of patients resected for colorectal liver metastasis, and a margin of ≥1 mm is needed to achieve the best possible outcome.

## Teaser

This national study examines how resection margins of 0.0 mm, 0.1–0.9 mm and ≥1 mm influence overall survival in patients resected for colorectal liver metastasis in an era of modern perioperative chemotherapy, analysing data from the national registries SweLiv and SCRCR. Despite modern chemotherapy the resection margin is still an important factor for the survival of patients resected for colorectal liver metastasis, and a margin of ≥1 mm is needed to achieve the best possible outcome.

## Introduction

Colorectal cancer (CRC) is the second most common cause of cancer-related death in Western countries and 25–30% of patients are diagnosed with colorectal liver metastases (CRLM)^1,2,3^. Modern treatment for CRLM including surgical resection and chemotherapy has resulted in a 5-year survival exceeding 50%^4,5^. Nowadays, resectability is defined as the possibility of performing a radical resection of all CRLM while leaving a sufficient functioning liver remnant^1,4^.

Numerous studies have described risk factors associated with poor survival after CRLM resection, namely advanced age, CRLM number and size, narrow resection margin, extrahepatic metastases, localization of the primary tumour, T- and N-stage of the primary tumour, synchronous metastases, high carcinoembryonic antigen (CEA) level, large perioperative blood loss or transfusions, progress on chemotherapy, Kirsten rat sarcoma virus oncogene (KRAS) or B-Raf proto-oncogene, serine/threonine kinase (BRAF) mutations, and perineural or vascular invasion of the tumour^6–9^. The tumour burden score (TBS) is a proposed comprehensive scoring method to evaluate metastatic burden and predict survival, derived from the size and number of metastases^10^. While the first proposed resection margin ‘standard’ was defined long ago as 10 mm, over time (and evidence accumulating) it has been reduced to ≥1 mm^1,11–18^. Recently, some studies have indicated that an R1 (microscopic tumour cells) resection margin may not affect survival, especially in the case of wild-type RAS tumours or in patients undergoing chemotherapy^19–22^.

Most studies on the significance of the CRLM resection margin have included patients resected before the modern era of systematic perioperative chemotherapy^11–13,17,23,24^. In Sweden, perioperative chemotherapy has been considered since the publication of the European Organisation for Research and Treatment of Cancer intergroup trial 40983 in 2008^23^.

The present study aimed to evaluate whether a microscopic resection margin of <1 mm, in a time of modern systematic administration of perioperative chemotherapy, impacts overall survival in patients with CRLM.

## Method

### Data assembly

Patients diagnosed with colorectal cancer in Sweden are prospectively registered in the Swedish Colorectal Cancer Registry (SCRCR), while patients undergoing liver surgery in Sweden are registered in the Swedish National Quality Registry for Liver, Bile Duct and Gallbladder Cancer (SweLiv). Those registries, when compared with the compulsory Swedish cancer registry, have a coverage of 99 and 97% respectively^5,25,26^. Data validity is high in both registries^26,27^. Ethical approval was obtained from the Swedish Ethical Review Authority (2019–02114). No further informed consent than already obtained from the SCRCR and SweLiv was needed for this study. During the study interval, liver surgery was performed in 11 institutions in Sweden. All patients radically resected for their primary colorectal cancer between 1 January 2009 and 31 December 2013 were identified in SCRCR. Patients were cross-linked with SweLiv, and patients also subjected to CRLM resections were included. Registry data were retrieved on 23 June 2020. Original pathology reports were scrutinized and exact margins validated in patients that had an unknown hepatic resection margin or a margin ≤2 mm reported in the registry, to allow for submillimetre analyses. Specimens were not reviewed.

Patients were excluded if they had surgery without liver resection or a macroscopic incomplete resection (R2), ablation techniques were used, pathology reports showed non-CRLM or the resection margin was unclear despite re-review of the original pathology report.

Characteristics of the primary colorectal cancer were extracted from the SCRCR, including tumour location, neoadjuvant chemotherapy and radiotherapy, TN-stage, histological tumour grading, vascular invasion, perineural invasion and operation date. Patient characteristics were retrieved from SweLiv, as well as characteristics of the metastases and the liver surgery, including age, sex, ASA score, intraoperative blood loss, number and size of CRLM, type of resection, involved Couinaud segments, presence of blue liver or steatotic liver, response to neoadjuvant chemotherapy (by radiological assessment), postoperative complications, staged surgeries, operation date, presence of extrahepatic metastases and histopathological resection margin.

### Definitions

Major hepatectomies were defined as resections of three or more connected Couinaud segments. Parenchymal-sparing surgery (PSS) was defined as non-anatomical resections, or anatomical resections of less than three Couinaud segments. Complications were categorized according to the Clavien–Dindo classification^28^. Major complications were defined as Clavien–Dindo ≥IIIa. Complications were registered in different ways in SweLiv during the study interval and were thus combined into a compound variable. The size of the largest metastasis was specified at the time of surgery. TBS was calculated from the size and number of metastases (TBS^2^ = (size of largest CRLM)^2^×(number of CRLM)^2^)^10^. Location of the primary tumour was defined as right-sided colon (caecum, ascending colon and transverse colon), left-sided colon (splenic flexure, descending colon and sigmoid colon) or rectal. Synchronous metastases were defined as metastases diagnosed prior to or within 3 months after the operation of the primary tumour. Extrahepatic disease was defined as any metastasis outside the liver at the time of surgery or having previous or planned surgery for extrahepatic disease. Response to neoadjuvant chemotherapy was defined as either radiological response or progress of the metastatic tumour burden in the liver. OS was calculated from the date of liver surgery until the date of death of any cause. Median follow-up time was estimated from the time of liver surgery until death of any cause, loss to follow-up or the end of the study (23 June 2020). In patients with repeated hepatectomies, only data from the first CRLM procedure was considered. In patients with staged hepatectomies, OS was calculated from the second surgery. The largest size and number of CRLM, the highest complication rating, the greatest blood loss and the narrowest resection margin from each of the staged surgeries were used for the analyses in patients with staged hepatectomies.

Patients were categorized into three groups according to the resection margin: 0 mm, 0.1–0.9 mm and ≥1 mm.

### Statistical analyses

Data are presented as median and interquartile range (i.q.r.) or proportions (%), as relevant. OS was illustrated with Kaplan–Meier curves and compared using the log-rank method. The 1-, 3- and 5-year survival rates were estimated and compared. The hazard ratios (HR) were calculated using a Cox proportional hazard model and are presented with 95% confidence intervals (c.i.). The proportional hazards assumption was tested by Schoenfeld residuals and was found to respect the assumption for the first 5 years. Univariable and multivariable analyses were performed. In the multivariable analyses, variables considered to be clinically relevant and those with a *P* < 0.100 in the univariable analysis, were tested for independent effects on survival, without stepwise selection. To avoid multicollinearity only TBS, and not number or size of CRLM, was used in multivariable analysis. A separate multivariable analysis on patients that received neoadjuvant chemotherapy was performed.

Missing data were analysed for systematic patterns and differences. Since none were found, missing data were assumed to be missing at random. Multiple imputation was performed using R software, version 4.1.0, 2021. Ten data sets were imputed. All variables later used in the multivariable analysis, except the outcome variable, follow-up time and resection margin, were used in the imputation model. Analyses using imputed data sets are presented in the results. Analyses using complete cases are presented in the *Supplementary material*.

Results were considered statistically significant at *P* < 0.05. Statistical analyses were performed using SPSS version 28.0, 2017 (IBM, Armonk, New York, USA) and R-software, version 4.1.0, 2021^29^.

## Results

**Fig. 1:**
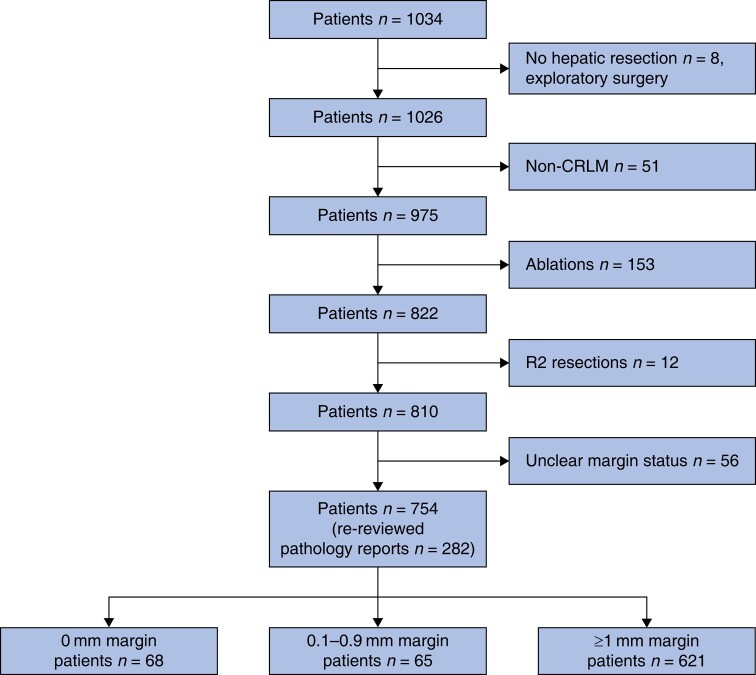
Flow chart

Between January 2009 and December 2013, 1034 patients were identified. After exclusion of 280 patients, 754 patients remained for analysis, *Fig. 1*. In all, 133 (17.6%) patients had a resection margin of <1 mm, and the median follow-up time was 5.4 years, *Table 1*. Some 456 patients died during follow-up.

Patients were excluded if they had surgery without liver resection or a macroscopic incomplete resection (R2), ablation techniques were used, pathology reports showed non-CRLM or the resection margin was unclear despite re-review of the original pathology report. CRLM, colorectal liver metastases.

**Table 1:** Patient and treatment characteristics in the cohort

Variable	Missing data (%)	0 mm (*n* = 68)	0.1–0.9 mm (*n* = 65)	1 mm+ (*n* = 621)	Total (*n* = 754)
**Patient**					
Age (years), median (i.q.r.)	0.1	64 (56.25–69)	68 (61–72)	65 (58–73)	66 (58–72)
Sex, male	0.0	38 (55.9)	42 (64.6)	368 (59.3)	448 (59.4)
ASA score ≥III	0.9	13 (19.1)	16 (24.6)	124 (20.0)	153 (20.3)
‘Blue liver’/SOS	2.7	24 (35.3)	13 (20.0)	117 (18.8)	154 (20.4)
Steatotic liver	2.0	16 (23.5)	11 (16.9)	84 (13.5)	111 (14.7)
**CRLM**					
Number of metastases >3	1.5	24 (35.3)	20 (30.8)	114 (18.4)	158 (21.3)
Size of largest tumour ≥5 cm	8.5	11 (16.2)	11 (16.9)	92 (14.8)	114 (15.1)
**TBS**	8.6				
<3		23 (33.8)	15 (23.1)	236 (38.0)	274 (36.3)
3–9		32 (47.1)	38 (58.5)	305 (49.1)	375 (49.7)
>9		9 (13.2)	5 (7.7)	26 (4.2)	40 (5.3)
Bilobar distribution of metastasis	0.9	36 (52.9)	32 (49.2)	201 (32.4)	269 (35.7)
**Response to chemotherapy**	1.9				
No chemo		18 (26.9)	19 (29.2)	212 (34.9)	249 (33.6)
Response/stable		47 (70.1)	45 (69.2)	384 (63.2)	476 (64.3)
Progress		2 (3.0)	1 (1.5)	12 (2.0)	15 (2.0)
Extrahepatic metastasis	0.5	10 (14.7)	4 (6.2)	40 (6.4)	54 (7.2)
Metachronous metastases	0.1	17 (25.0)	26 (40.0)	213 (34.3)	256 (34.0)
**Intraoperative**					
Anatomical resection	2.4	27 (39.7)	21 (32.3)	273 (44.0)	321 (42.6)
Major resection	2.4	30 (44.1)	28 (43.1)	231 (37.2)	289 (38.3)
Blood loss ≥1000 ml	1.9	28 (41.2)	26 (40.0)	195 (31.4)	249 (33.0)
Parenchymal-sparing resection	2.4	38 (55.9)	34 (52.3)	375 (60.4)	447 (59.3)
Staged liver surgery	0.0	8 (11.8)	1 (1.5)	10 (1.6)	19 (2.5)
Liver first	0.0	22 (32.4)	17 (26.2)	170 (27.4)	209 (27.7)
**Postoperative complications after liver surgery**	17.3				
None		29 (42.6)	41 (63.1)	443 (71.3)	513 (68.0)
Minor		9 (13.2)	8 (12.3)	56 (9.0)	73 (9.7)
Major		10 (14.7)	6 (9.2)	41 (6.6)	57 (7.6)
Subsequent liver surgery	0.0	12 (17.6)	19 (29.2)	94 (15.1)	125 (16.6)
**Primary tumour**					
CRC location	0.0				
Right		23 (33.8)	14 (21.5)	142 (22.9)	179 (23.7)
Left		29 (42.6)	26 (40.0)	251 (40.4)	306 (40.6)
Rectum		16 (23.5)	25 (38.5)	228 (36.7)	269 (35.7)
N-status positive	0.1	46 (67.6)	46 (70.8)	400 (64.4)	492 (65.3)
T-stage ≥3	0.0	59 (86.8)	53 (81.5)	546 (87.9)	658 (87.3)
Differentiation grade poor	3.9	10 (14.7)	6 (9.2)	86 (13.8)	102 (13.5)
Vascular invasion	7.6	32 (47.1)	26 (40.0)	238 (38.3)	296 (39.3)
Perineural invasion	18.9	25 (36.8)	17 (26.2)	126 (20.3)	168 (22.3)
Neoadjuvant chemotherapy*	0.0	24 (35.3)	23 (35.4)	196 (31.6)	243 (32.2)
Neoadjuvant radiotherapy*	0.0	10 (14.7)	24 (36.9)	189 (30.4)	223 (29.6)

Values are *n* (%) unless otherwise stated. TBS, tumour burden score; SOS, sinusoidal obstruction syndrome; CRLM, colorectal liver metastases; CRC, colorectal cancer; i.q.r., interquartile range; *before bowel surgery.

### Univariable analyses

**Fig. 2:**
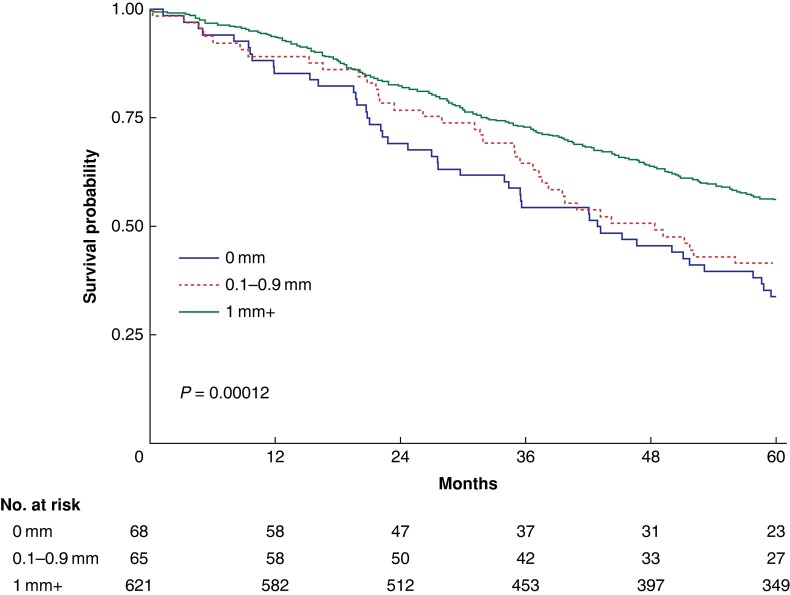
**Survival curves.** Overall survival (OS) of patients undergoing hepatic resections for CRLM, stratified by the resection margin. The median OS was 42 (95% c.i. 31 to 53) months in the 0 mm group, 48 (95% c.i. 35 to 62) months for the 0.1–0.9 mm group and 75 (95% c.i. 65 to 85) months for the ≥1 mm group. Compared using the log-rank method. CRLM, colorectal liver metastases.

The median OS was 42 (95% c.i. 31 to 53) months in the 0 mm group, 48 (95% c.i. 35 to 62) months in the 0.1–0.9 mm group and 75 (95% c.i. 65 to 85) months in the ≥1 mm group, *P* < 0.001. In the whole cohort, the median OS was 65 (95% c.i. 57 to 73) months. Survival curves are presented in *Fig. 2*. The 1-, 3- and 5-year OS rates are presented in *Table 2*. The univariable analyses of association with OS are presented in *Table 3*.

**Table 2:** Estimated 1-, 3- and 5-year overall survival rates

Margin status	1 year	3 years	5 years
0 mm	85.1 (76.5,93.6)	53.7 (41.8,65.7)	32.8 (21.6,44.1)
0.1–0.9 mm	89.2 (81.7,96.8)	64.6 (53.0,76.2)	41.5 (29.6,53.5)
≥1 mm	93.7 (91.8,95.6)	72.9 (69.5,76.2)	56.2 (52.2,60.1)
Total cohort	92.6 (90.7,94.4)	70.5 (67.3,73.8)	52.9 (49.3,56.4)

Values are % (95% c.i.). 1-, 3- and 5-year survival rates in relation to resection margin.

**Table 3:** Risk factors associated with overall survival

Variable	Univariable model		Multivariable model	
	HR (95% c.i.)	*P*	HR (95% c.i.)	*P*
**Resection margin**				
0 mm	1.700 (1.267,2.281)	**<0.001**	1.413 (1.030,1.939)	**0**.**032**
0.1–0.9 mm	1.577 (1.169,2.126)	**0.003**	1.399 (1.025,1.910)	**0**.**034**
≥1 mm	1.0 (Ref.)		1.0 (Ref.)	
**Patient**				
Age (years)	1.011 (1.002,1.021)	**0**.**020**	1.014 (1.004,1.025)	**0.009**
Sex, male	1.031 (0.855,1.244)	0.750	1.031 (0.848,1.253)	0.761
ASA ≥ III	1.326 (1.064,1.654)	**0**.**012**	1.160 (0.919,1.464)	0.211
‘Blue liver’/SOS	1.034 (0.824,1.297)	0.772		
Steatotic liver	0.973 (0.748,1.266)	0.840		
**CRLM**				
No. of metastases >3	1.391 (1.115,1.735)	**0**.**003**		
Size of largest tumour ≥5 cm	1.737 (1.368,2.206)	**<0**.**001**		
**TBS**				
<3	1.0 (Ref.)		1.0 (Ref.)	
3–9	1.289 (1.048,1.584)	**0.016**	1.151 (0.907,1.460)	0.247
>9	2.621 (1.795,3.828)	**<0.001**	1.882 (1.237,2.865)	**0**.**003**
Bilobar	1.369 (1.134,1.652)	**0**.**001**	1.219 (0.983,1.512)	0.071
**Response to chemotherapy**				
No chemo	0.867 (0.734,1.095)	0.285	1.108 (0.879,1.397)	0.386
Response/stable	1.0 (Ref.)		1.0 (Ref.)	
Progress	1.950 (1.094,3.476)	**0.024**	2.032 (1.109,3.723)	**0**.**022**
Extrahepatic metastasis	1.231 (0.875,1.730)	0.233	1.056 (0.727,1.534)	0.777
Metachronous metastasis	0.862 (0.707,1.051)	0.142	0.921 (0.743,1.140)	0.448
**Intraoperative characteristics**				
Anatomical resection	1.077 (0.892,1.300)	0.442		
Major resection	1.362 (1.129,1.643)	**0**.**001**	1.292 (1.041,1.603)	**0.020**
Blood loss ≥1000 ml	1.215 (1.003,1.472)	**0**.**047**	1.053 (0.849,1.306)	0.638
Staged liver surgery	2.641 (1.601,4.354)	**<0**.**001**	1.611 (0.885,2.932)	0.119
Liver first	0.994 (0.810,1.221)	0.956		
**Postoperative complications**				
None	1.0 (Ref.)		1.0 (Ref.)	
Minor	1.109 (0.807,1.524)	0.522	1.010 (0.729,1.399)	0.952
Major	1.724 (1.246,2.386)	**0.001**	1.559 (1.083,2.244)	**0**.**018**
Subsequent liver surgery	1.151 (0.912,1.453)	0.235	1.165 (0.908,1.495)	0.229
**Primary tumour**				
CRC location				
Right	1.0 (Ref.)		1.0 (Ref.)	
Left	0.817 (0.646,1.033)	0.091	0.942 (0.735,1.208)	0.638
Rectum	0.872 (0.687,1.107)	0.261	1.043 (0.808,1.347)	0.744
N-status positive	1.838 (1.493,2.263)	**<0**.**001**	1.538 (1.230,1.923)	**<0.001**
T-stage ≥3	1.246 (0.931,1.667)	0.139	1.079 (0.788,1.477)	0.637
Differentiation grade poor	1.298 (1.001,1.684)	**0**.**049**	1.153 (0.874,1.521)	0.315
Vascular invasion	1.628 (1.345,1.970)	**<0**.**001**	1.296 (1.048,1.602)	**0.017**
Perineural invasion	1.658 (1.337,2.056)	**<0**.**001**	1.415 (1.131,1.771)	**0.002**

Results from univariable and multivariable Cox regression models. Analysis of factors associated with OS among patients resected for CRLM. The multivariable model is analysed using imputed data sets, based on 10 iterations. Bold values are significant *P* < 0.050. TBS, tumour burden score; CRLM, colorectal liver metastases; SOS, sinusoidal obstruction syndrome; CRC, colorectal cancer; OS, overall survival; Ref, reference category.

### Multivariable analyses

A narrow resection margin was found to be a risk factor with an independent association with poor OS, *Table 3*. The 0 mm group had a HR of 1.413 (95% c.i. 1.030 to 1.939) and the 0.1–0.9 mm group 1.399 (95% c.i. 1.025 to 1.910), compared with the ≥1 mm group. Increasing age and a high TBS >9 was associated with survival.

Major surgery and major complications were associated with poor OS. In the multivariable analysis, neither blood loss >1000 ml nor staged liver surgery were found to correlate significantly with OS. Progress on chemotherapy was associated with poor OS, with a HR of 2.032 (95% c.i. 1.109 to 3.723). Not receiving neoadjuvant chemotherapy had a similar HR as response to chemotherapy. Analysing patients that received neoadjuvant chemotherapy separately, the resection margin was still independently associated with OS in multivariable analysis, with a HR of 1.742 (95% c.i. 1.195 to 2.541) for 0 mm and 1.622 (95% c.i. 1.123 to 2.343) for 0.1–0.9 mm, *Table 4*.

**Table 4:** Risk factors associated with overall survival in the subset of patients that received neoadjuvant chemotherapy

Variable	Univariable model		Multivariable model	
	HR (95% c.i.)	*P*	HR (95% c.i.)	*P*
**Resection margin**				
0 mm	1.794 (1.272,2.530)	**0.001**	1.742 (1.195,2.541)	**0**.**004**
0.1–0.9 mm	1.662 (1.169,2.363)	**0.005**	1.622 (1.123,2.343)	**0**.**010**
≥1 mm	1.0 (Ref.)		1.0 (Ref.)	
**Patient**				
Age (years)	1.012 (0.999,1.025)	0.062	1.013 (1.000,1.027)	0.056
Sex, male	1.039 (0.827,1.305)	0.742	1.002 (0.792,1.268)	0.988
ASA ≥ III	1.250 (0.933,1.675)	0.135	1.041 (0.760,1.425)	0.804
‘Blue liver’/SOS	1.034 (0.824,1.297)	0.772		
Steatotic liver	0.973 (0.748,1.266)	0.840		
**CRLM**				
No. of metastases >3	1.391 (1.115,1.735)	**0**.**003**		
Size of largest tumour ≥5 cm	1.737 (1.368,2.206)	**<0**.**001**		
**TBS**				
<3	1.0 (Ref.)		1.0 (Ref.)	
3–9	1.175 (0.902,1.531)	0.232	1.081 (0.804,1.454)	0.604
>9	2.496 (1.616,3.857)	**<0.001**	2.208 (1.391,3.506)	**0**.**001**
Bilobar	1.276 (1.017,1.600)	**0**.**035**	1.126 (0.871,1.457)	0.364
**Response to chemotherapy**				
Response/stable	1.0 (Ref.)		1.0 (Ref.)	
Progress	1.954 (1.096,3.484)	**0.023**	2.088 (1.121,3.889)	**0**.**020**
Extrahepatic metastasis	0.967 (0.632,1.480)	0.878	0.968 (0.606,1.547)	0.892
Metachronous metastasis	0.866 (0.661,1.134)	0.296	0.899 (0.679,1.191)	0.458
**Intraoperative characteristics**				
Anatomical resection	1.077 (0.892,1.300)	0.442		
Major resection	1.416 (1.128,1.777)	**0**.**003**	1.429 (1.111,1.837)	**0.005**
Blood loss ≥1000 ml	1.135 (0.904,1.426)	0.275	1.099 (0.857,1.409)	0.457
Staged liver surgery	2.348 (1.371,4.019)	**0**.**002**	1.718 (0.910,3.241)	0.095
Liver first	0.994 (0.810,1.221)	0.956		
**Postoperative complications**				
None	1.0 (Ref.)		1.0 (Ref.)	
Minor	1.051 (0.692,1.595)	0.815	0.888 (0.580,1.361)	0.585
Major	1.517 (1.019,2.259)	**0.040**	1.407 (0.935,2.118)	0.101
Subsequent liver surgery	1.239 (0.945,1.626)	0.121	1.276 (0.954,1.707)	0.101
**Primary tumour**				
CRC location				
Right	1.0 (Ref.)		1.0 (Ref.)	
Left	0.856 (0.634,1.155)	0.308	1.063 (0.770,1.469)	0.710
Rectum	0.842 (0.620,1.145)	0.272	1.107 (0.791,1.550)	0.552
N-status positive	1.835 (1.414,2.381)	**<0**.**001**	1.623 (1.219,2.161)	**0.001**
T-stage ≥3	1.375 (0.963,1.965)	0.080	1.130 (0.769,1.660)	0.534
Differentiation grade poor	1.278 (0.930,1.756)	0.130	1.214 (0.866,1.701)	0.259
Vascular invasion	1.714 (1.356,2.167)	**<0**.**001**	1.436 (1.111,1.857)	**0.006**
Perineural invasion	1.486 (1.142,1.933)	**0**.**003**	1.317 (1.011,1.717)	**0.042**

Results from univariable and multivariable Cox regression models. Analysis of factors associated with OS among the subset of patients that received neoadjuvant chemotherapy before being resected for CRLM. *n* = 491. The multivariable model was analysed using imputed data sets, based on 10 iterations. Bold values are significant *P* < 0.050. TBS, tumour burden score; CRLM, colorectal liver metastases; SOS, sinusoidal obstruction syndrome; CRC, colorectal cancer; OS, overall survival; Ref., reference category.

The T-stage of the primary tumour had no association with OS, but positive nodal status was strongly associated with reduced OS. The location of the primary tumour was not found to correlate to OS, and neither was the timing of the diagnosis of the metastases. Perineural and vascular invasion of the primary tumour were found to have a negative impact on survival.

The multivariable analysis of complete cases (*n* = 438) did not demonstrate individual association of the resection margin with OS, *Table S1*.

## Discussion

In a national population-based cohort of patients who underwent liver resection of CRLM, a surgical margin of <1 mm impacted overall survival. In multivariable analyses age, progress on chemotherapy, high TBS, major hepatic surgery, major postoperative complications, positive nodal status of the primary tumour, histological vascular invasion and perineural invasion were also independent risk factors of poor OS.

Studies investigating the impact of the surgical resection margin on OS have shown varying results. While some large cohort and propensity-matched analyses demonstrated that a resection margin <1 mm was associated with poor OS^12,17,30^, others failed^15,22,31^. There are conflicting results as to whether neoadjuvant chemotherapy influences the association between survival and the resection margin^19,20,22^. In this study, neoadjuvant chemotherapy was related to OS only in patients with tumour progression during chemotherapy. Patients not receiving chemotherapy had no increased risk compared with those who responded, probably due to the fact that neoadjuvant chemotherapy is often reserved for less favourable tumour status. The effect on OS of neoadjuvant chemotherapy in resectable situations remains uncertain^23,32,33^. A recent randomized study found no improvement in OS after adjuvant chemotherapy in patients with primarily resectable disease^34^. Perioperative chemotherapy was liberally used in this study cohort and the resection margin was found to be associated with OS both in the subgroup of patients receiving neoadjuvant chemotherapy and the entire cohort.

Tumour biology and advanced metastatic disease have been proposed to override the effects of the resection margin^31,35^. Factors related to the tumour biology and the metastatic tumour burden were also found to influence OS in this study, but together with the margin status. The primary tumour biology was associated with OS by N-status, vascular invasion and perineural invasion^6,8,9,36,37^. High TBS, bilobar disease, major hepatectomy and staged liver surgery were associated with worse OS in the univariable analysis, and these characteristics were found in the groups with a narrow margin. Advanced metastatic disease may increase the risk of having a narrow resection margin and could thus be a confounder. However, both in the full cohort and in the subgroup that received neoadjuvant chemotherapy, the multivariable analyses showed that major hepatectomy and high TBS were independently associated with poor OS, together with a narrow margin. Hence, a clear margin status ≥1 mm improves survival along with tumour biology and must also be achieved in patients who have received neoadjuvant chemotherapy.

A strength of this study was the large national cohort from two national validated registries SweLiv and SCRCR with high coverage, allowing wide generalizability and a real-life picture of patients having resections for CRLM^5,25^. Only patients undergoing radical bowel surgery were included, addressing the possible confounder of residual primary tumour tissue. During this study time interval modern perioperative chemotherapy regimens were used, making the study relevant in a modern setting. An analysis of the submillimetre margin in patients with a narrow margin was reported. Multiple imputation was used in the analyses, minimizing the bias of discarding relevant data, improving the precision and the validity of the analyses. The retrospective design was a limitation of the study. Registry data are also limited, lacking some important information such as genetic mutations, CEA levels, histological lymph vessel invasion, adjuvant chemotherapy, recurrence-free survival and whether narrow margins were parenchymal or vascular. Further limitations were the inclusion of only the first liver resection data, not the following.

Future studies on resection margin should aim to evaluate if adjuvant chemotherapy can improve OS in patients with a narrow margin. Genetic mutations such as KRAS status should also be included in the analyses, as this may impact the results. Furthermore, the impact of resection margins in repeated hepatectomies should be studied specifically. In conclusion, the present study shows that the resection margin is an independent risk factor of reduced OS in CRLM patients, and surgeons should strive for a margin of ≥1 mm.

## Supplementary Material

zrae035_Supplementary_Data

## Data Availability

Data are available through the Swedish national quality registries SweLiv and SCRCR following appropriate approval; anonymized data can be shared upon request.
