# Supplementary material for: Importance of resection margin after resection of colorectal liver metastases in the era of modern chemotherapy: population-based cohort study
Source: BJS Open. 2024 May 8;8(3):zrae035. doi: 10.1093/bjsopen/zrae035 (PMC11078257; doi:10.1093/bjsopen/zrae035)
Supplement: zrae035_Supplementary_Data [file zrae035_supplementary_data.docx]

**Title:** Importance of resection margin after resection of colorectal liver metastases in the era of modern chemotherapy: population-based cohort study

**Authors:** Emil Östrand (MD)^1,2^, Jenny Rystedt (MD, PhD)^1,2^, Jennie Engstrand (MD, PhD) ^3,4^, Petter Frühling (MD, PhD) ^5,6^, Oskar Hemmingsson (MD, PhD)^7^, Per Sandström (MD, PhD) ^8,9^, Malin Sternby Eilard (MD, PhD)^10,11^, Bobby Tingstedt (MD, PhD)^1,2^, Pamela Buchwald (MD, PhD)^12,13^

^1^Department of Surgery, Skåne University Hospital, Lund, Sweden

^2^Department of Clinical Sciences Lund, Lund University, Sweden

^3^Division of Surgery, Karolinska University Hospital, Stockholm, Sweden

^4^Department of Clinical Science, Intervention and Technology (CLINTEC), Karolinska Institutet, Stockholm, Sweden

^5^Department of Surgery, Akademiska University Hospital, Uppsala, Sweden

^6^Department of Surgical Sciences, Uppsala University, Sweden

^7^Department of Surgical and perioperative Sciences, Umeå University, Sweden

^8^Department of Surgery, Linköping University hospital, Linköping, Sweden

^9^Department of Clinical and Experimental Medicine Sciences, Linköping University, Sweden

^10^Department of Transplantation and Liver Surgery, Sahlgrenska University hospital, Göteborg, Sweden

^11^Department of Clinical Sciences, Sahlgrenska Academy, University of Gothenburg, Sweden

^12^Department of Surgery, Skåne University Hospital, Malmö, Sweden

^13^Department of Clinical Sciences Malmö, Lund University, Sweden

**Corresponding author:** Emil Östrand MD, Department of Surgery, Skåne University Hospital, SE‑22 185 Lund, Sweden. ORCID ID: 0000-0001-7621-9541. Phone: 046-172383 E-Mail: [emil.ostrand@med.lu.se](mailto:emil.ostrand@med.lu.se)

**Supplementary Materials - Index**

| **Supplementary Figures and Tables** |  |
| --- | --- |
| Table S1. Overall Survival. Results of multivariable cox-regression, Complete Cases-model. | *pag. 3* |
| Table S2. Risk factors associated with overall survival in the subset of patients that did receive neoadjuvant chemotherapy. Results from multivariable cox-regression, complete cases model. | *pag. 4* |
|  |  |

**Supplementary Figures and Tables**

**Table S1. Risk factors associated with overall survival. Results of multivariable cox-regression, Complete Cases-model**

| n=438 | | Multivariable  Complete cases-model | |
| --- | --- | --- | --- |
| Variable | | HR (95% CI) | p-value |
|  | |  |  |
| Margin | |  |  |
| Marginal categories | |  |  |
| 0mm | | 1.101 (0.710-1.710) | 0.667 |
| 0.1-0.9mm | | 1.192 (0.788-1.801) | 0.406 |
| ≥1mm | | 1.0 (Ref.) |  |
|  | |  |  |
| Patient | |  |  |
| Age, years | | 1.022 (1.007-1.037) | **0.004** |
| Gender, male | | 1.162 (0.886-1.524) | 0.279 |
| ASA≥3 | | 1.056 (0.776-1.437) | 0.727 |
|  | |  |  |
| CRLM | |  |  |
| TBS | |  |  |
|  | <3 | 1.0 (Ref.) |  |
|  | 3-9 | 1.236 (0.913-1.673) | 0.170 |
|  | >9 | 2.550 (1.414-4.598) | **0.002** |
| Bilobar | | 1.066 (0.795-1.430) | 0.668 |
| Response to chemotherapy | |  |  |
|  | No chemo | 1.066 (0.789-1.440) | 0.677 |
|  | Response | 1.0 (Ref.) |  |
|  | Progress | 2.583 (1.021-6.537) | **0.045** |
| Extrahepatic metastasis | | 1.467 (0.899-2.391) | 0.125 |
| Metachronous metastasis | | 0.859 (0.646-1.141) | 0.294 |
|  | |  |  |
| Intraoperative | |  |  |
| Major surgery | | 1.165 (0.869-1.561) | 0.307 |
| Blood loss ≥1000 ml | | 0.954 (0.708-1.285) | 0.755 |
| Staged liver surgery | | 0.760 (0.267-2.166) | 0.608 |
|  | |  |  |
| Postoperative | |  |  |
| Complications | |  |  |
|  | None | 1.0 (Ref.) |  |
|  | Minor | 1.022 (0.694-1.505) | 0.913 |
|  | Major | 1.478 (0.955-2.287) | 0.080 |
| Subsequent liver surgery | | 1.184 (0.837-1.676) | 0.340 |
|  | |  |  |
| Primary tumour | |  |  |
| CRC Location | |  |  |
|  | Right | 1.0 (Ref.) |  |
|  | Left | 1.017 (0.721-1.434) | 0.923 |
|  | Rectum | 1.053 (0.739-1.503) | 0.774 |
| N-stage positive | | 1.833 (1.336-2.516) | **<0.001** |
| T-stage ≥3 | | 1.055 (0.686-1.622) | 0.807 |
| Differentiation grade poor | | 1.426 (1.001-2.031) | **0.049** |
| Vascular invasion | | 1.356 (1.027-1.791) | **0.032** |
| Perineural invasion | | 1.477 (1.099-1.986) | **0.010** |
|  | |  |  |

Multivariable Complete cases-analysis of factors associated with OS among patients resected for CLRM. n=438.

TBS= Tumour Burden Score. CRLM=Colorectal Liver Metastases. ASA= American Society of Anaesthesiologists. CRC=Colorectal cancer.

**Table S2. Risk factors associated with overall survival in the subset of patients that did receive neoadjuvant chemotherapy. Results from multivariable cox-regression, complete cases model.**

|  | | Multivariable model | |
| --- | --- | --- | --- |
| Variable | | HR (95% CI) | p-value |
|  | |  |  |
| Margin | |  |  |
| Marginal categories | |  |  |
|  | 0mm | 1.403 (0.802-2.454) | 0.235 |
|  | 0.1-0.9mm | 1.426 (0.874-2.325) | 0.155 |
|  | ≥1mm | 1.0 (Ref.) |  |
|  | |  |  |
| Patient | |  |  |
| Age, years | | 1.016 (0.997-1.035) | 0.104 |
| Gender, male | | 1.171 (0.840-1.634) | 0.351 |
| ASA≥3 | | 1.085 (0.715-1.646) | 0.702 |
| “Blue liver” / SOS | |  |  |
| Steatotic liver | |  |  |
|  | |  |  |
| CRLM | |  |  |
| No of metastasis >3 | |  |  |
| Size of largest tumour ≥5cm | |  |  |
| TBS | |  |  |
|  | <3 | 1.0 (Ref.) |  |
|  | 3-9 | 1.160 (0.782-1.722) | 0.461 |
|  | >9 | 2.772 (1.470-5.224) | **0.002** |
| Bilobar | | 0.942 (0.664-1.339) | 0.741 |
| Response to chemotherapy | |  |  |
|  | Response / stable | 1.0 (Ref.) |  |
|  | Progress | 2.517 (0.968-6.546) | 0.058 |
| Extrahepatic metastasis | | 1.378 (0.730-2.602) | 0.323 |
| Metachronous metastasis | | 0.879 (0.600-1.287) | 0.507 |
|  | |  |  |
| Intraoperative characteristics | |  |  |
| Anatomical resection | |  |  |
| Major resection | | 1.320 (0.939-1.855) | 0.110 |
| Blood loss ≥1000 ml | | 0.866 (0.611-1.227) | 0.419 |
| Staged liver surgery | | 0.760 (0.251-2.302) | 0.627 |
| Liver first | |  |  |
|  | |  |  |
| Postoperative | |  |  |
| Complications | |  |  |
|  | None | 1.0 (Ref.) |  |
|  | Minor | 0.871 (0.517-1.468) | 0.604 |
|  | Major | 1.399 (0.828-2.363) | 0.209 |
| Subsequent liver surgery | | 1.277 (0.835-1.954) | 0.259 |
|  | |  |  |
| Primary tumour | |  |  |
| CRC Location | |  |  |
|  | Right | 1.0 (Ref.) |  |
|  | Left | 1.009 (0.650-1.565) | 0.970 |
|  | Rectum | 1.020 (0.638-1.630) | 0.935 |
| N-status positive | | **2.137 (1.399-3.263)** | **<0.001** |
| T-stage ≥3 | | 1.049 (0.607-1.812) | 0.864 |
| Differentiation grade poor | | 1.303 (0.837-2.029) | 0.242 |
| Vascular invasion | | 1.478 (1.046-2.087) | **0.027** |
| Perineural invasion | | 1.225 (0.861-1.741) | 0.260 |
|  | |  |  |

Univariable and multivariable analysis of factors associated with OS among the subset of patients that received neoadjuvant chemotherapy before being resected for CLRM. The model is analysed using complete cases only. TBS= Tumour Burden Score. CRLM=Colorectal Liver Metastases. ASA= American Society of Anaesthesiologists. SOS=Sinusoidal obstruction syndrome. CRC=Colorectal cancer.
